# Supplementary material for: TamGen: drug design with target-aware molecule generation through a chemical language model
Source: Nat Commun. 2024 Oct 29;15:9360. doi: 10.1038/s41467-024-53632-4 (PMC11522292; doi:10.1038/s41467-024-53632-4)
Supplement: Supplementary file 1 — Supplementary Information [file 41467_2024_53632_MOESM1_ESM.pdf]

TamGen: Drug Design with Target-aware Molecule Generation through a Chemical Language Model

This document contains the following supplementary materials:

- Supplementary figures from Fig. S1 to Fig. S11;
- Supplementary tables from Table S1 to Table S4;
- Supplementary notes;
- Synthesis path of novel compounds.

1 Supplementary figures

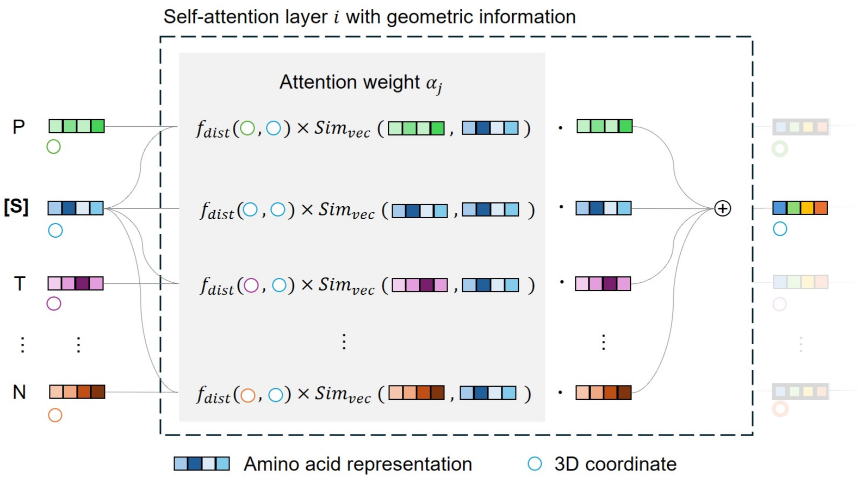

Fig. S1: **Details of the self-attention mechanism with geometric information used in the protein encoder.** For each amino acid representation in layer i, the attention weight α's is calculated as the product of the amino acid representation similarity and negative geometric distances between pairs of amino acids (i.e., $\exp(-\text{distances}^2/\tau)$ where τ is a hyperparameter). The output of layer i is then derived from the sum of the α's multiplied by the amino acid representation.

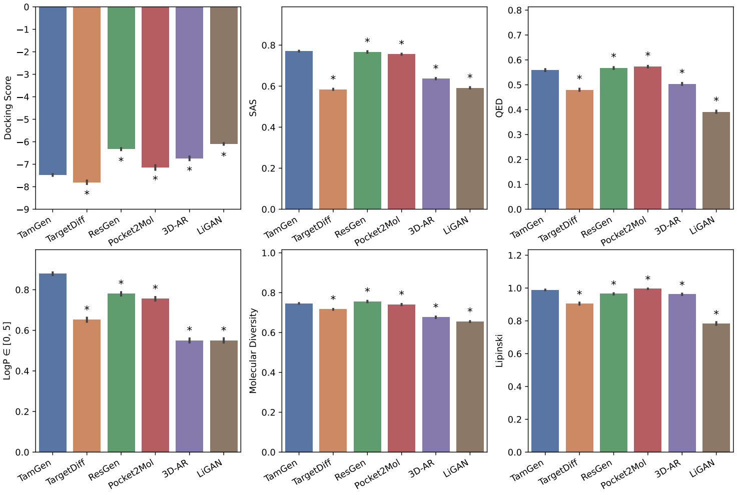

Fig. S2: **Docking scores, SAS, QED, LogP$\in [0, 5]$, Molecular Diversity and Lipinski of various generative drug design methods in relation to the CrossDocked2020 task.** Bar, mean values. Error bar, 95% confidence interval. p-values between results of TamGen and alternate approaches are calculated with Mann–Whitney U test. Star(*) indicates a p-value smaller than 1e-6. The original data used for plotting can be found in Table S1.

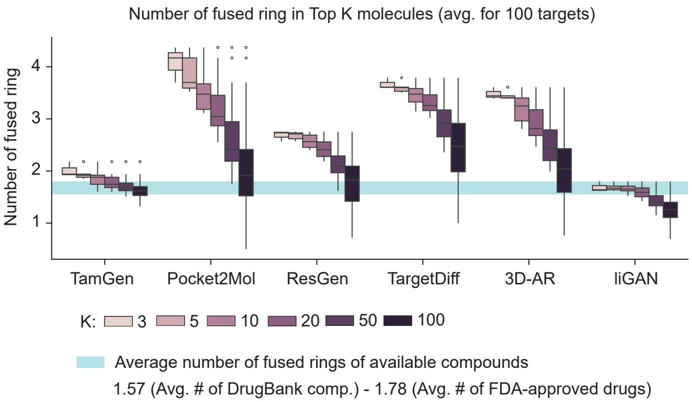

Fig. S3: **The distribution of fused ring numbers in compounds generated by different methods.** K represents the number of compounds having top-K docking scores against each target protein. Center line, median; box limits, upper and lower quartiles; whiskers, 1.5x interquartile range; points, outliers.

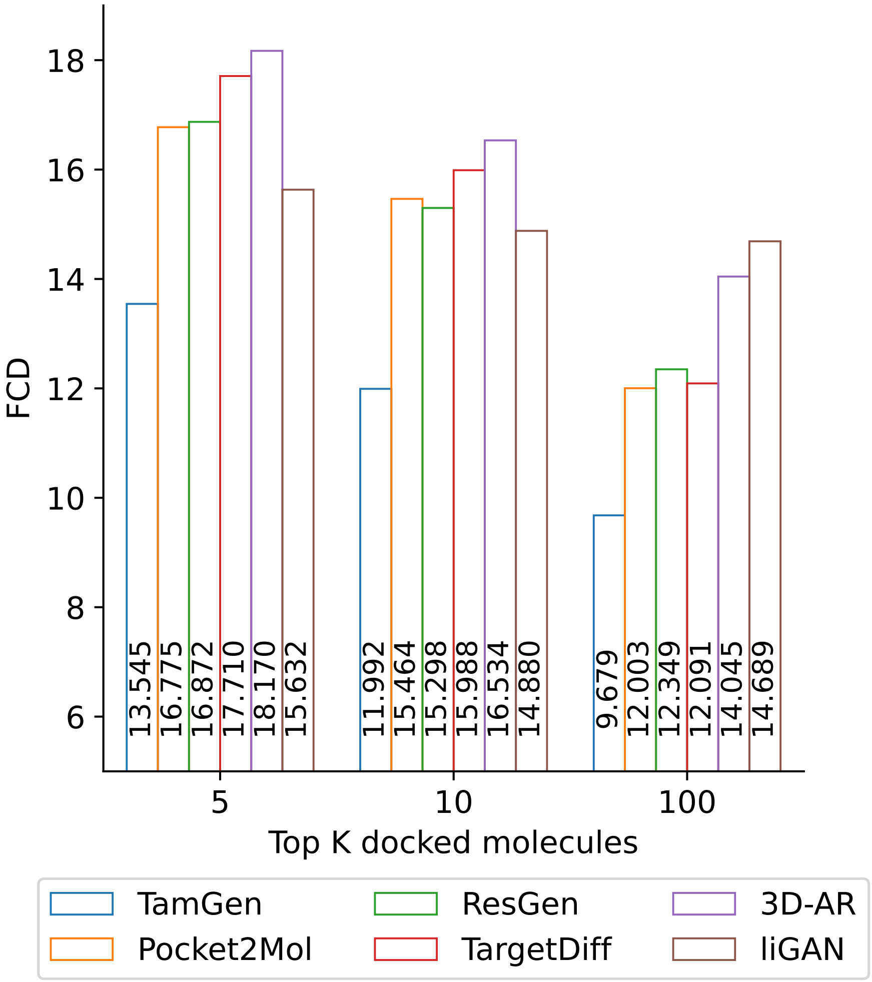

Fig. S4: **The Fréchet ChemNet Distance (FCD) similarity** [1] **scores between FDA-approved drugs and compounds produced by different methods.** FCD is a metric that quantifies the distributional dissimilarities between two compound sets, referred to as group A and group B. In this context, group A comprises all FDA-approved drugs, while group B includes compounds generated through various methods. A lower FCD score indicates a closer distribution of the generated compounds to the FDA approved drugs, signifying their similarity. TamGen demonstrates the capability to generate compounds that are most akin to FDA-approved drugs, as evidenced by the lowest FCD scores.

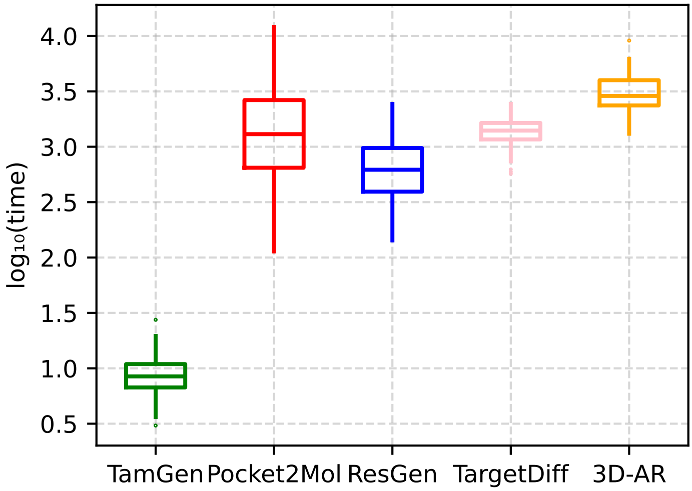

Fig. S5: **TamGen significantly outperforms alternate methods on running time.** The y-axis is scaled using a logarithm base 10.

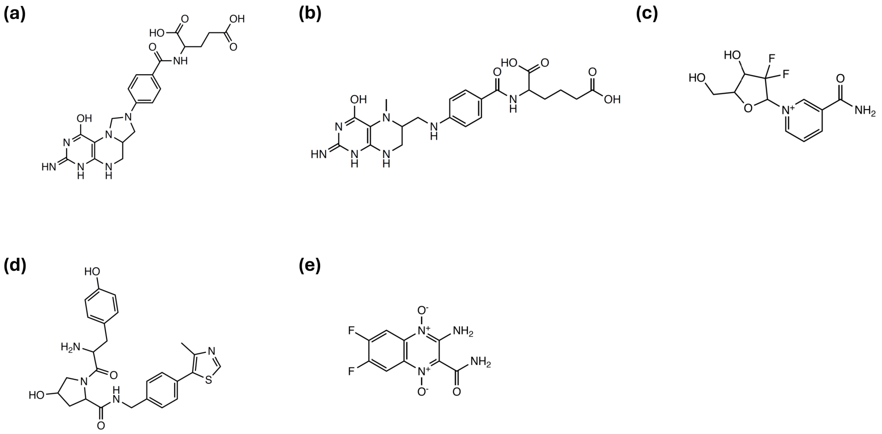

Fig. S6: **Seeding compounds for Stage 2 generation. (a-d)** The four seeding compounds selected from the first round; **(e)**: One example of the experimental selected compound.

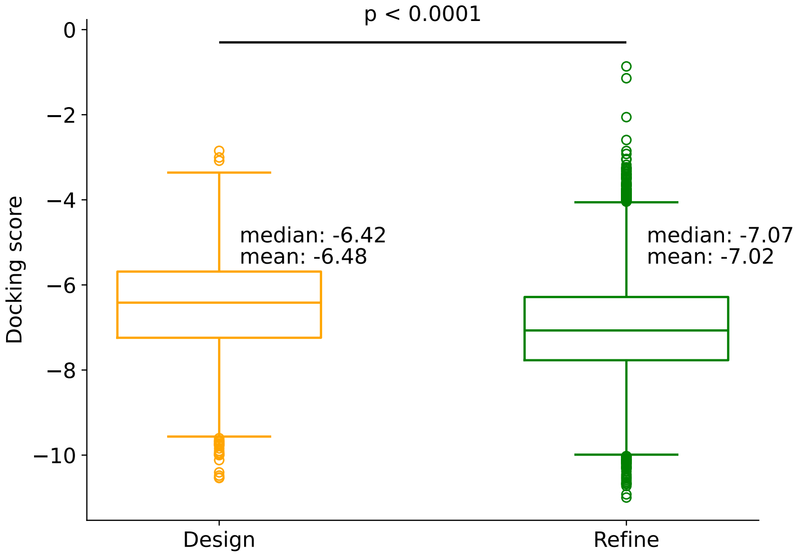

Fig. S7: **Distribution of docking scores for generated compounds against ClpP**. Center line, median; box limits, upper and lower quartiles. p-value is calculated with Mann–Whitney U test (`scipy.stats.mannwhitneyu`).

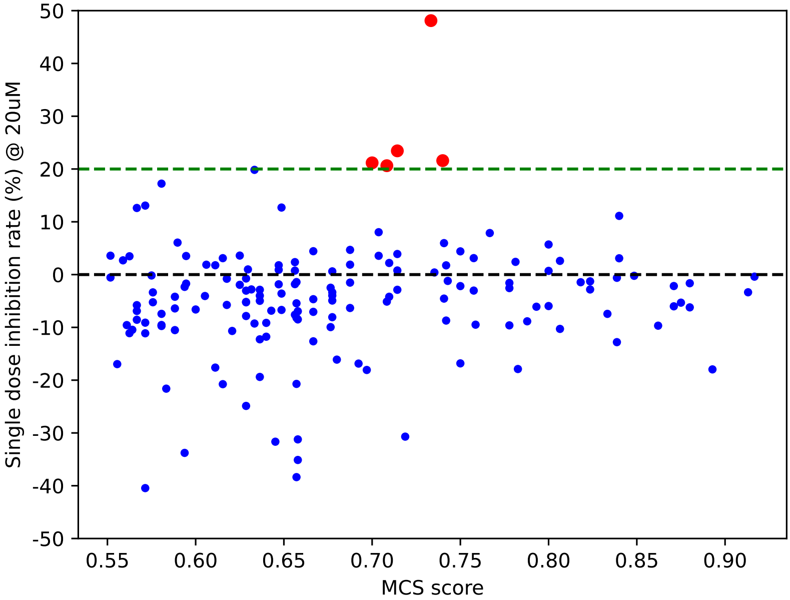

Fig. S8: **Inhibition rate of the 159 library search analogs relative to Bortezomib**. All compounds were evaluated at the concentration of 20 µM. The dashed line indicates the threshold for analog selection. x-axis: Maximum Common Substructure (MCS) similarity scores. The mean values and standard derivations of the MCS scores of the 159 selected compounds are 0.681 and 0.090, respectively. The Spearman correlation between MCS similarity scores and inhibition rates is 0.158. See Methods for details.

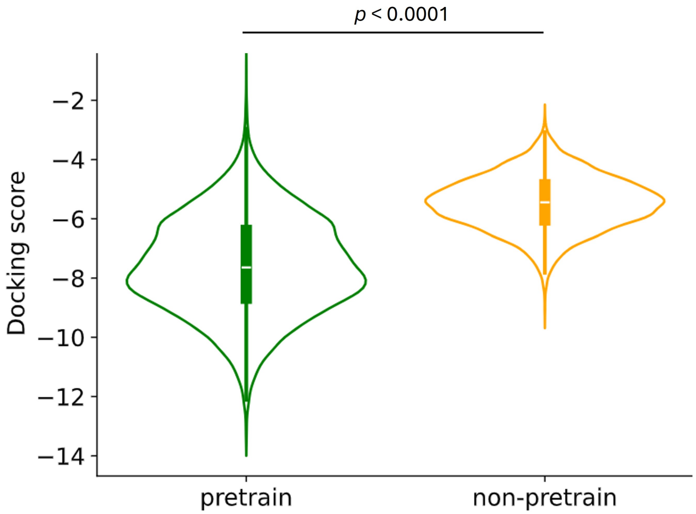

(a) The violin plot illustrates the docking scores of pretrained and non-pretrained compound decoder. Pretrained decoder shows a significant improvement compared to non-pretrained decoder. p-value is calculated with Mann–Whitney U test (`scipy.stats.mannwhitneyu`).

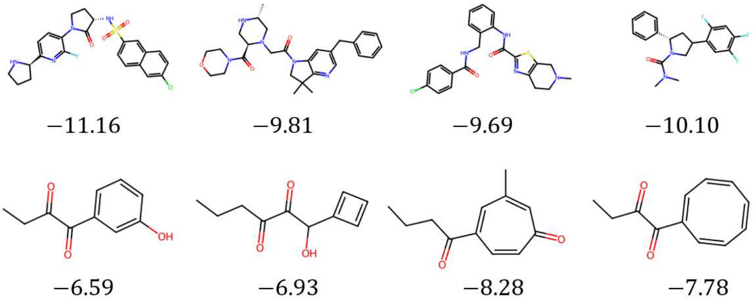

(b) Case study of the generated compounds. The top/bottom rows are the compounds generated by pre-trained / non-pretrained compound generators respectively. The corresponding docking scores for each compound are displayed under their respective structures. Each column corresponds to the same target. The compounds are visualized using RDKit.

Fig. S9: **Ablation study indicates that pre-training is essential for molecule generation of the compound decoder.**

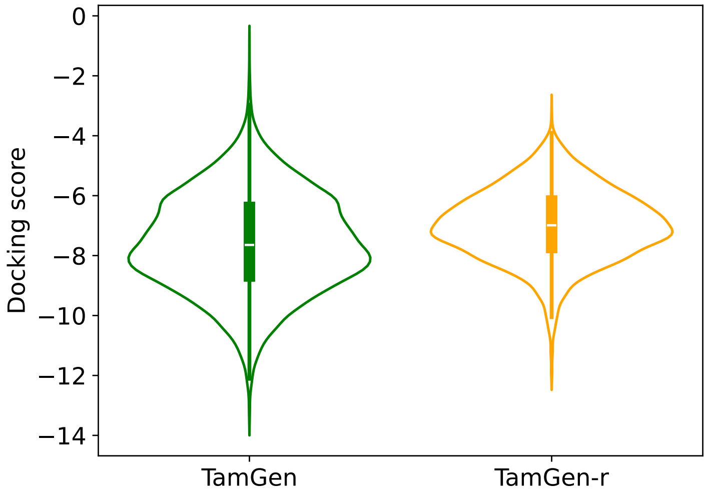

Fig. S10: **The violin plot illustrates the docking scores of TamGen and TamGen-r, which is finetuned on the randomized protein-ligand pairs.** The docking scores of TamGen is better than TamGen-r with $p < 0.00001$, where the p-value is calculated with Mann–Whitney U test (`scipy.stats.mannwhitneyu`)

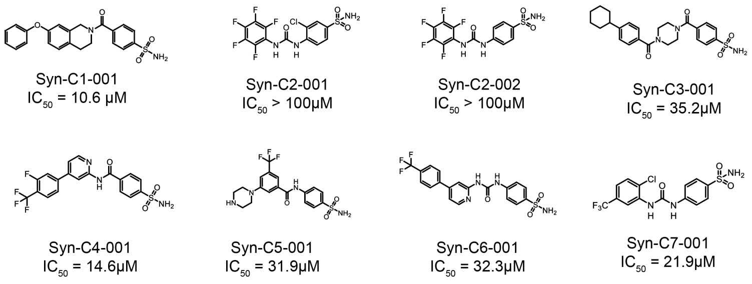

Fig. S11: The novel compounds generated by TamGen and their IC_{50} values.

2 Supplementary tables

Model \ Metric(pval)	Vina Dock (↓)	QED (↑)	SAS (↑)	Diversity (↑)	LogP∈ [0, 5]	Lipinski (↑)
liGAN	-6.099 (0)	0.392 (0)	0.592 (0)	0.655 (0)	55.0% (0)	78.4% (0)
3D-AR	-6.746 (2e-160)	0.503 (6e-91)	0.637 (0)	0.698 (1e-152)	55.0% (0)	96.2% (1e-32)
Pocket2Mol	-7.152 (1e-53)	0.573 (9e-6)	0.756 (4e-13)	0.741 (2e-38)	75.5% (9e-112)	99.5% (7e-8)
TargetDiff	-7.802 (1e-33)	0.480 (1e-148)	0.585 (0)	0.717 (5e-86)	65.2% (4e-302)	90.5% (2e-149)
ResGen	-6.326 (0)	0.567 (9e-7)	0.767 (4e-32)	0.756 (8e-192)	78.0% (3e-77)	96.5% (1e-28)
TamGen	-7.475 (-)	0.559 (-)	0.771 (-)	0.747 (-)	87.9% (-)	98.8% (-)

Table S1: Compilation of performance statistics for all methods across various evaluation metrics used for illustration in Fig. 2a. Mean values are reported in the upper part of each cell and p-values calculated using the Mann-Whitney U test are reported in parentheses.

ID	PubChem	Commercial library source	IC_{50} (µM)
Analog-001	2810424	Maybridge Screening Collection	17.2
Analog-002	45503904	Life Chemicals HTS Compound Collection	19.9
Analog-003	2813477	Maybridge Screening Collection	10.1
Analog-004	160268	reframeDB	19.6
Analog-005	4851126	Selleck PFZ	1.9

Table S2: Resources of the analogue compounds. The index of the compounds, PubChem CID, Commercial library source and IC_{50} values are summarized.

	Vina Dock (↓)	QED (↑)	SAS (↑)	Diversity (↑)	LogP∈ [0, 5]	Lipinski (↑)
TamGen	-7.475	0.559	0.771	0.747	87.9%	98.8%
TamGen w/o `dist_attn`	-6.943	0.543	0.773	0.761	86.6%	98.4%
TamGen w/o `coord_aug`	-6.588	0.564	0.774	0.766	75.1%	97.3%

Table S3: Compilation of performance statistics for TamGen and two variants: (i) TamGen w/o `dist_attn` denotes the variant where the distance-aware attention is removed; (ii) TamGen w/o `coord_aug` is the variant where the coordinate augmentation is removed.

Methods	Docking (↑)	LogP	QED (↑)	SA (↓)
AlphaDrug w/o MCTS	8.5	4.0	0.5	2.7
AlphaDrug (max)	11.6	5.2	0.4	2.7
TamGen	10.1	3.9	0.5	2.6

Table S4: Comparative Analysis with AlphaDrug [2]. We provided 10 compounds per receptor following the protocols of AlphaDrug and conducted evaluations using AlphaDrug's metrics. An upward arrow (↑) indicates that a higher value is advantageous, whereas a downward arrow (↓) suggests that a lower value is preferable. "AlphaDrug w/o MCTS" denotes a variant of AlphaDrug that operates without the Monte Carlo Tree Search (MCTS) optimization (i.e., the "LT+BS10" in Table 1 of [2]).

3 Supplementary notes

3.1 Compare GPT-based decoder with RNN decoder

To verify the effectiveness of the GPT-based decoder, we replace the decoder part in TamGen to the LSTM layer, which is the widely used RNN architecture. We explore the number of decoder layers L from $1, 2, 4$ and found that setting $L = 1$ achieves the best performance under our implementation. The results are shown in Table S5. In terms of docking scores, the Transformer-based model significantly outpeforms the RNN-based model with p-value$< 10^{-10}$.

	Vina Dock (↓)	QED (↑)	SAS (↑)	Diversity (↑)	LogP$\in [0, 5]$	Lipinski (↑)
TamGen	-7.475	0.559	0.771	0.747	87.9%	98.8%
LSTM	-6.364	0.607	0.791	0.725	96.7%	99.9%

Table S5: Comparison between our method and the RNN-based encoder and decoder.

3.2 Similarities between generated compounds with molecules in libraries

To verify the similarity between our generated compounds and their corresponding analogs in established databases, we computed the similarity scores of our generated compounds and the analogs against three databases: (1) the dataset used for training our model (briefly denoted as "training set"); (2) the Drug Repurposing Hub with FDA-approved drugs (denoted as "FDA approved"); (3) a randomly selected subset of 1 million compounds from Enamine, which is a commercial compound library (denoted as "Enamine"). We focused on determining both the top-3 and top-10 similarity metrics.

More specifically, for each ligand l, whether originating from the generation or analogs, we computed the Morgan fingerprint similarity between l and every ligand in the reference database (training set, FDA approved, or Enamine). Subsequently, we calculated the average of the top-3 and top-10 fingerprint similarity scores. Finally, the mean scores are visualized by using violin plot in Fig. S12.

We have the following observations:

1. Regardless of whether we consider the top-3 or top-10 similarity metrics, the generated compounds exhibit the highest similarity to the training set. This outcome is expected since a machine learning model is designed to capture the distribution characteristics of the data. The similarity to Enamine, the commercial chemical library, ranks second. This correlation is logical given the extensive size and diversity of the commercial library, which encompasses a broad spectrum of potentially beneficial compounds.
2. In terms of top-3 and top-10 accuracy, the analogs display the greatest similarity to the commercial library Enamine. This is attributable to the fact that the high-throughput screening (HTS) library used in our experiments is encompassed within the larger commercial library. The similarity scores do not reach the maximum value of 1 consistently due to our utilization of only a subset of Enamine (1 million compounds) to manage computational costs. Nonetheless, the pattern is clear.

We additionally present the structural diversity among the 296 chosen compounds, the 159 analog compounds, and the remaining pool of 8,069 compounds. The structural diversity between any two compounds c_1 and c_2 is defined as "$1 - \texttt{fingerpint\_similarity}(c_1, c_2)$". This calculation of structural diversity is performed for each pair of distinct compounds within their respective groups (i.e., the 296 chosen compounds, the 159 analog compounds, and the remaining pool of 8,069 compounds). The outcomes of this analysis are in Figure S12(c). From the data, we can see that the set of 296 selected compounds exhibits the highest level of structural diversity, succeeded by the analog compounds. This observation underscores the efficiency of generative techniques in spanning a broader expanse of chemical space. Nevertheless, it is also noted that the diversity within the selected compounds is relatively moderate, with a majority of diversity values falling below 40%.

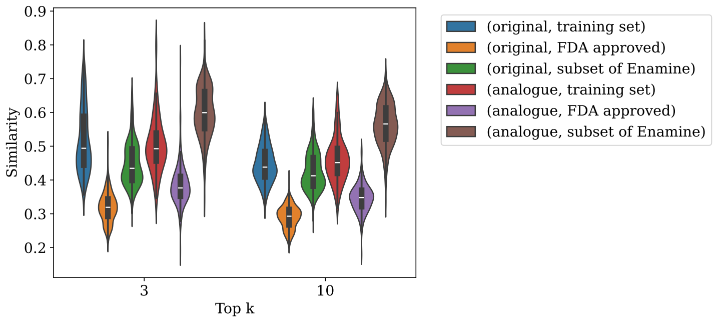

(a) Fingerprint similarity of the selected 296 compounds and their 159 analog compounds to the training set, FDA-approved drugs, and a subset of the Enamine library.

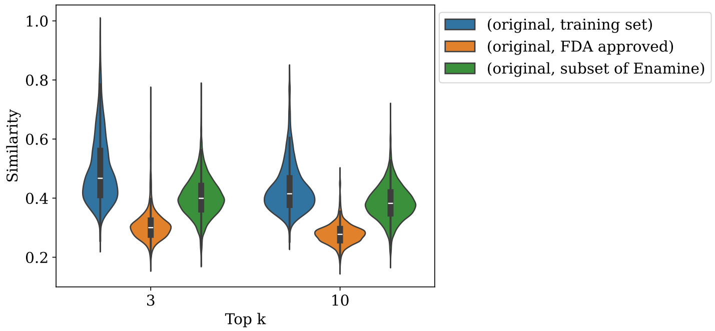

(b) Fingerprint similarity of the remaining 8069 compounds to the training Set, FDA-approved Drugs, and a subset of the Enamine library.

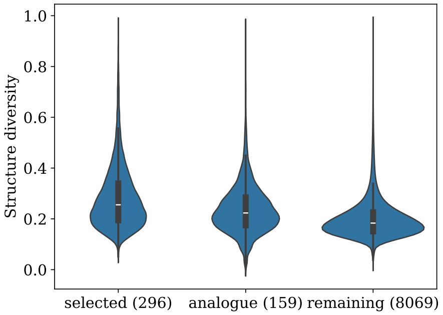

(c) Pairwise structure diversity of the 296 selected compound, the 159 analogue compounds and the remaining 8069 generated compounds.

Fig. S12: Fingerprint similarity of the generated compounds and their analogue compounds to established compound libraries. The analysis is partitioned into the 296 compounds and their corresponding analogues (depicted in subfigure (a)) and the remaining compounds (depicted in subfigure (b)). The structure diversity of the selected 296 compounds, the 159 analogue compounds and the remaining 8069 compounds are shown in subfigure (c).

Id for subfigure (a)
1. (original, training set)
2. (original, FDA approved)
3. (original, subset of Enamine)
4. (analogue, training set)
5. (analogue, FDA approved)
6. (analogue, subset of Enamine)

Id for subfigure (b)
1. (original, training set)
2. (original, FDA approved)
3. (original, subset of Enamine)

Id for subfigure (c)
1. selected (296)
2. analogue (159)
3. remaining (8069)

** indicates p-value < 10^{-6}

p-values for subfigure (a)

id1, id2	Top-3	Top-10
1,2	**	**
1,3	**	**
1,4	0.418	0.089
1,5	**	**
1,6	**	**
2,3	**	**
2,4	**	**
2,5	**	**
2,6	**	**
3,4	**	**
3,5	**	**
3,6	**	**
4,5	**	**
4,6	**	**
5,6	**	**

p-values for subfigure (b)

id1, id2	Top-3	Top-10
1,2	**	**
1,3	**	**
2,3	**	**

p-values for subfigure (c)

id1, id2	p-value
1,2	**
1,3	**
2,3	**

(d) p-values of the statistics from subfigure (a) to (c).

Fig. S12: (d) shows the p-values calculated using the Mann-Whitney U test between pairwise samples.

To address the observed limitations in diversity, future work could involve refining the generative model by incorporating diversity-promoting objectives or by expanding the training dataset to include a wider variety of chemical structures. Additionally, employing diversity-oriented selection criteria during the compound selection phase could help in capturing a broader representation of the model's generative capabilities.

3.3 Ablation study to the pocket size

To evaluate the association between protein pocket size and performance, we categorized the pockets based on the numbers of residues (denoted as $S(\mathbf{x})$) as follows:

1. If $S(\mathbf{x}) < 40$, pocket $\mathbf{x}$ is a defined as a small pocket;
2. If $S(\mathbf{x}) \in [40, 60)$, pocket $\mathbf{x}$ is a defined as a medium pocket;
3. If $S(\mathbf{x}) \geq 60$, pocket $\mathbf{x}$ is a defined as a large pocket.

We computed the mean reciprocal rank (MRR) values for the generated compounds corresponding to each pocket size category. The results (reported in Fig. S13) demonstrate that TamGen consistently performs well across diverse protein pocket sizes. In addition, most approaches, except ResGen, tend to maintain consistent performance irrespective of pocket size.

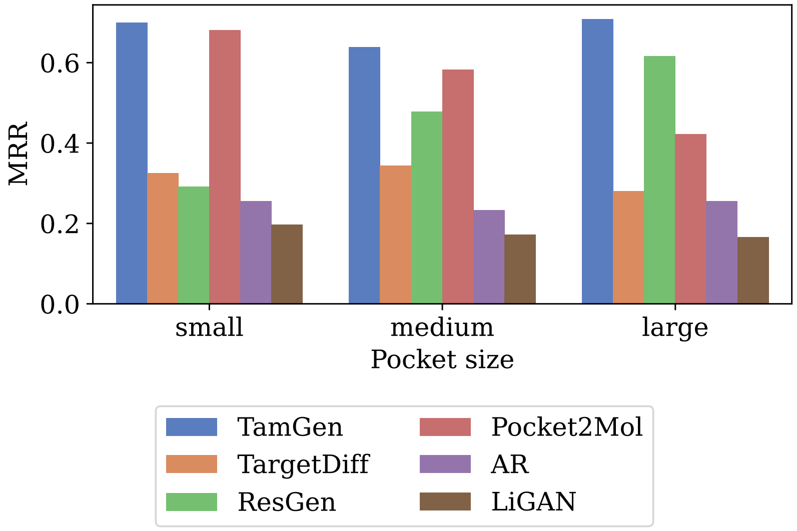

Fig. S13: **MRR of the generated compounds of different methods with respect to pocket sizes.**

3.4 Correlation between LogP and Lipinski's rule of five

LogP is one of the criteria in Lipinski's rule of five (Ro5). Therefore, to rigorously evaluate the potential overlap between Ro5 and LogP values, both of which were used in Mean Reciprocal Rank (MRR) calculation in our study, we undertook a focused analysis by randomly sampling 1 million compounds from the PubChem database and determining their compliance with Ro5 as well as their LogP values. We then assessed the association between these two properties.

Given that compliance with Ro5 is a binary outcome, we employed the Point-biserial correlation coefficient (denoted as r_{pb}), a measure of the strength of association between a continuous variable (LogP) and a binary variable (Ro5 compliance), to quantify the relationship. We use the python package `scipy.stats.pointbiserialr` to calculate this metric.

For the total sample of 1 million compounds, the r_{pb} between Ro5 compliance and LogP values was found to be 0.146. Further, we filtered compounds whose LogP values fall within the range of [0, 5] (indicating high drug-likeness) and found an r_{pb} of 0.044 between Ro5 compliance and LogP values for these compounds. Both r_{pb} values suggest a poor correlation between the two metrics, implying that considering both Ro5 and LogP simultaneously for MRR calculation allows for a more comprehensive evaluation of the model outputs from different perspectives.

3.5 Selection of seeding compound with low activity against Mtb ClpP for refinement

We selected three additional compounds with relatively low activity against Mtb ClpP (100-200 µM) for our model. This decision was guided by the principles of fragment-based drug discovery [3]. In fragment-based drug discovery, the initial focus is on identifying small, structurally simple molecules known as "fragments", which often exhibit low affinity when binding to target sites. Despite their low initial activity, these fragments offer significant advantages due to their simplicity and small size. For example, they can serve as strategic starting points in the drug discovery process, allowing for incremental improvements through iterative cycles of design, synthesis, and testing. This aligns well with the refinement capabilities of TamGen. In this context, the inclusion of these additional compounds allows us to demonstrate the utility of our method in optimizing structurally simple fragments into more potent and selective inhibitors.

Supplementary References

[1] Preuer, K., Renz, P., Unterthiner, T., Hochreiter, S., Klambauer, G.: Fréchet chemnet distance: A metric for generative models for molecules in drug discovery. Journal of Chemical Information and Modeling **58**(9), 1736–1741 (2018). https://doi.org/10.1021/acs.jcim.8b00234. PMID: 30118593

[2] Qian, H., Lin, C., Zhao, D., Tu, S., Xu, L.: AlphaDrug: protein target specific de novo molecular generation. PNAS Nexus **1**(4), 227 (2022). https://doi.org/10.1093/pnasnexus/pgac227

[3] Kirsch, P., Hartman, A.M., Hirsch, A.K.H., Empting, M.: Concepts and core principles of fragment-based drug design. Molecules **24**(23), 4309 (2019)

4. Synthesis path of novel compounds

Synthesis of Syn-C1-001 in the SI

The chemists responsible for the synthesis refer to the compound Syn-C1-001 by an internal code GDI-009209.

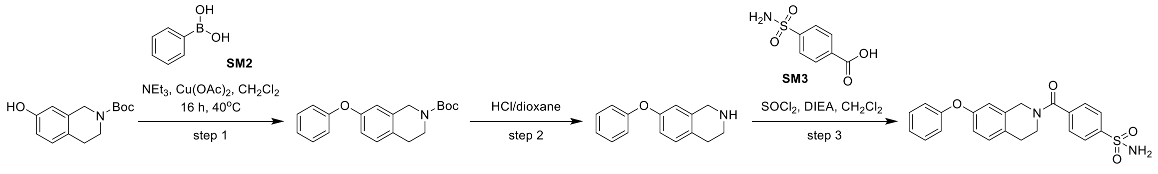

GDI-009209

4-(7-phenoxy-1,2,3,4-tetrahydroisoquinoline-2-carbonyl)benzenesulfonamide（GDI-009209）

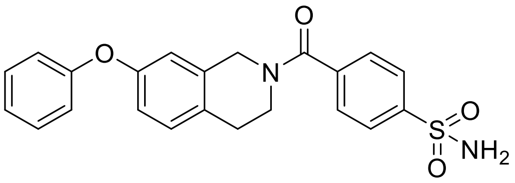

GDI-009209

^{1}H NMR (400 MHz, DMSO) δ 8.00 (t, *J* = 8.5 Hz, 2H), 7.68 – 7.57 (m, 2H), 7.39 – 7.27 (m, 2H), 7.18 (s, 1H), 7.10 (s, 1H), 7.00-6.83 (m, 4H), 4.84 (s, 1H), 4.53 (s, 1H), 3.99 (s, 1H), 3.62 (s, 1H), 2.96 (s, 1H), 2.87 (s, 1H).

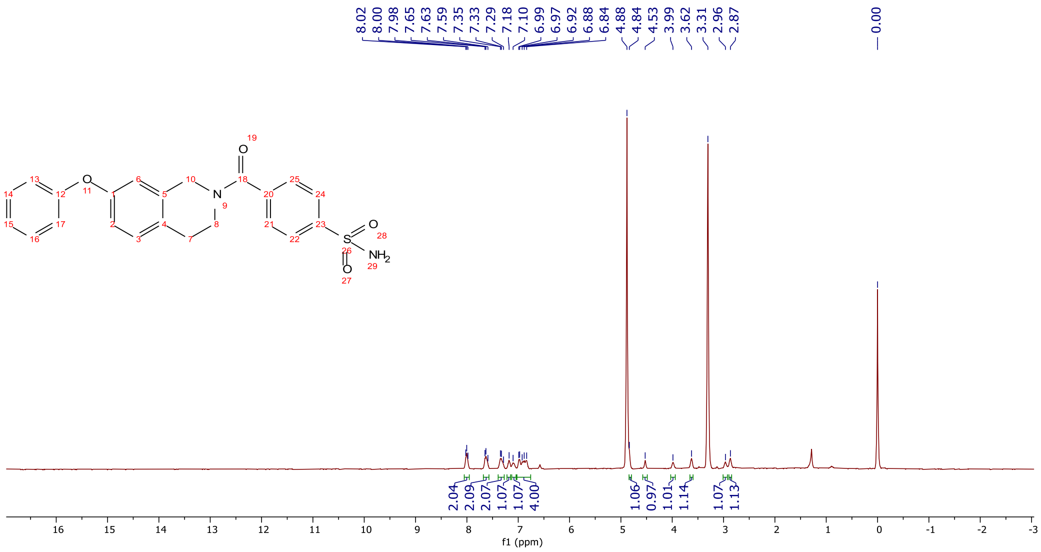

^{1}H NMR of **GDI-009209**

Synthesis of Syn-C2-001 in the SI

The chemists responsible for the synthesis refer to the compound Syn-C2-001 by an internal code GDI-009051.

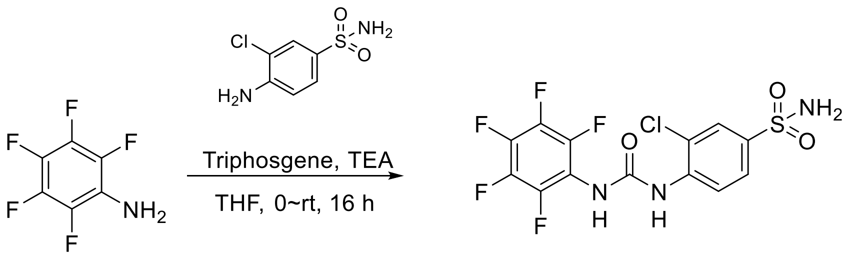

GDI-009051

Step 1: Synthesis of 3-chloro-4-(3-(perfluorophenyl)ureido)benzenesulfonamide (GDI-009051)

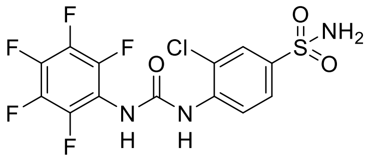

GDI-009051

To a solution of 2,3,4,5,6-pentafluoroaniline (1000.00 mg, 5.46 mmol), 4-amino-3-chlorobenzenesulfonamideb (1128.74 mg, 5.46 mmol) and TEA (1105.42 mg, 10.9242 mmol) in dry THF (10 mL) was added Triphosgene (810.44 mg, 2.73 mmol) dropwise at 0 ℃ under an atmosphere of N_2. After addition, the solution was stirred at 20 ℃ for 12 h. The final mixture was quenched with H_2O and extracted with EtOAc. The combined organic layers were washed with water and brine, dried with sodium sulfate, and concentrated under vacuum. The residue was purified by silica gel column chromatography (eluting with EtOAc/PE, 40% to 50%) to give 1-(2-chloro-4-sulfamoylphenyl)-3-(2,3,4,5,6-penta fluorophenyl)urea (500 mg, 22.02% yield) as a white solid.MS (ESI) m/z = 413.9 $[M+H]^+$

^{1}H NMR (400 MHz, d6-DMSO) δ 9.42 (s, 1H), 8.95 (s, 1H), 8.31 (d, J = 8.8 Hz, 1H), 7.88 (d, J = 2.0 Hz, 1H), 7.73 (dd, J = 8.6, 2.2 Hz, 1H), 7.40 (s, 2H).

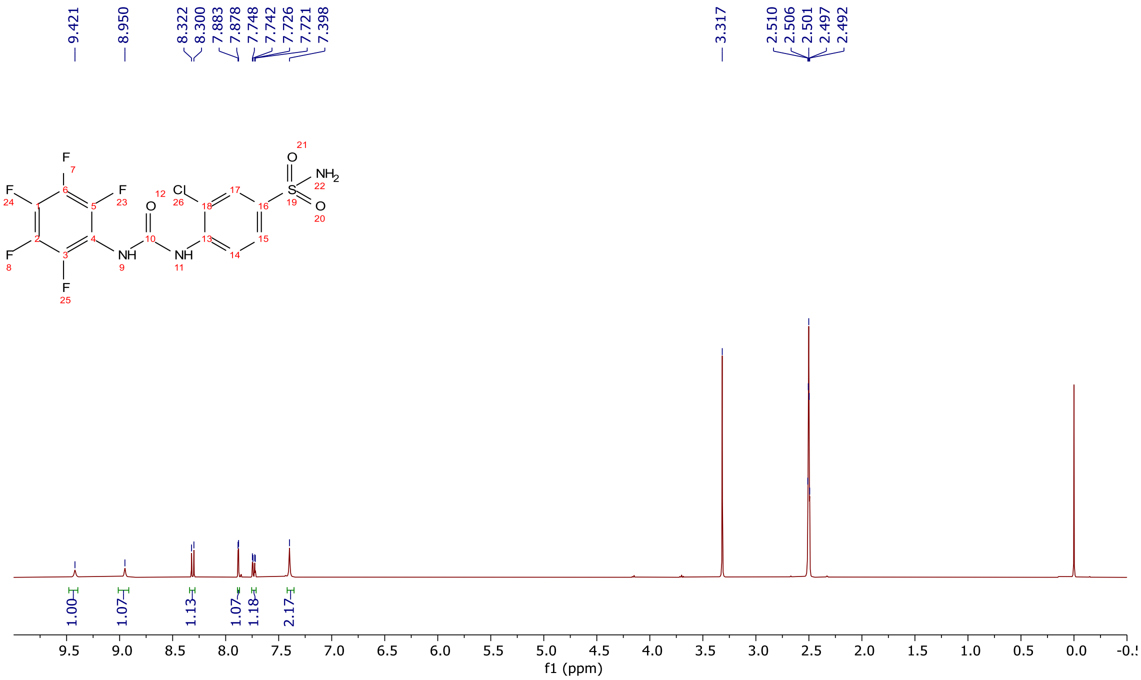

^{1}H NMR of **GDI-009051**

Synthesis of Syn-C2-002 in the SI

The chemists responsible for the synthesis refer to the compound Syn-C2-002 by an internal code GDI-009052.

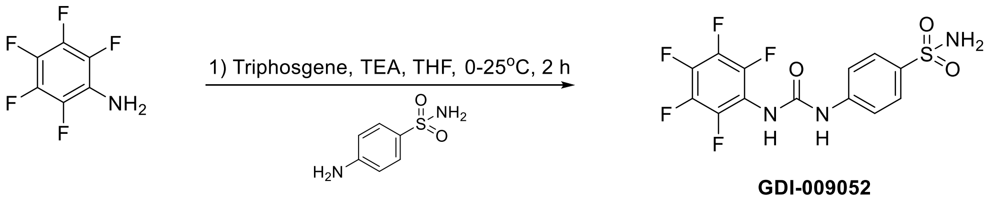

Step 1: Synthesis of 4-(3-(perfluorophenyl)ureido)benzenesulfonamide (*GDI-009052)*

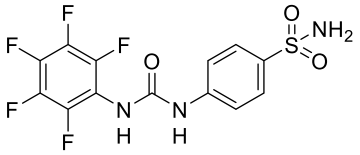

GDI-009052

To a solution of 2,3,4,5,6-pentafluoroaniline (500.00 mg, 2.73 mmol) and Triphosgene (221.00 mg, 0.82 mmol) in THF (6 mL) at 0°C was added TEA (829.00 mg, 8.19 mmol) dropwise and stirred at 25°C for 2 h. Then 4-aminobenzenesulfonamide (470.00 mg, 2.73 mmol) was added in one charge. The reaction mixture was stirred at 25°C for 4 h. The resulting mixture was quenching with NH_4Cl and concentrated. The residue was purified by prep-HPLC (Gemini 5 um C18 column, 150*21.2 mm, eluting with 30% to 90% MeCN/H_2O containing 0.1% FA) to afford 4-(3-(perfluorophenyl)ureido)benzene sulfonamide (76 mg, 0.20 mmol) as white solid.MS (ESI) m/z = 381.95 $[M+H]^+$

^{1}**H NMR** (400 MHz, d6-DMSO) δ 9.53 (s, 1H), 8.72 (s, 1H), 7.73 (d, J = 8.7 Hz, 2H), 7.61 (d, J = 8.8 Hz, 2H), 7.22 (s, 2H).

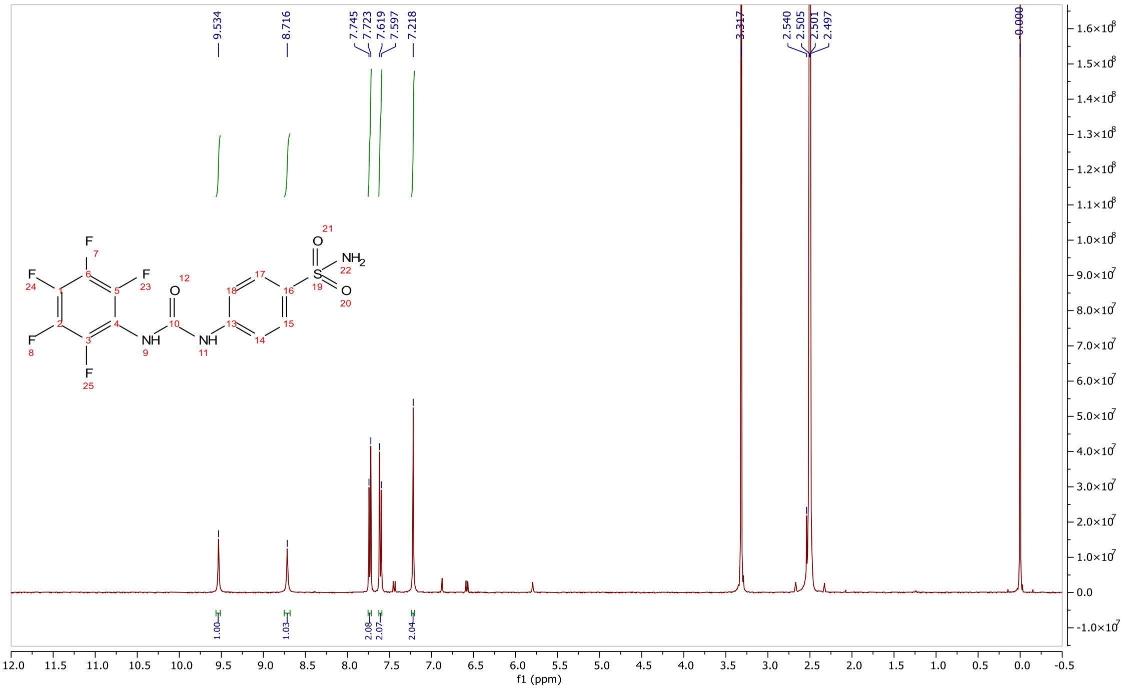

^{1}H NMR of **GDI-009052**

Synthesis of Syn-C3-001 in the SI

The chemists responsible for the synthesis refer to the compound Syn-C3-001 by an internal code GDI-009108.

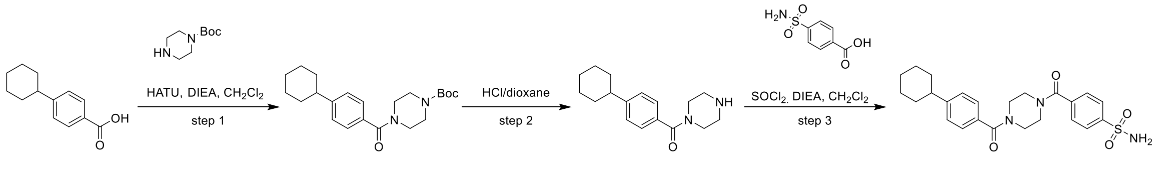

<u>Step 1: synthesis of tert-butyl 4-(4-cyclohexylbenzoyl)piperazine-1-carboxylate (B-1)</u>

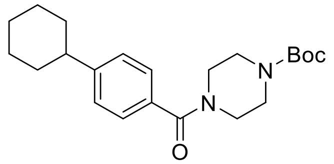

A mixture of 4-cyclohexylbenzoic acid (408.00 mg, 2.0 mmol), tert-butyl piperazine-1-carboxylate (372.00 mg, 2.0 mmol), HATU (1140.00 mg, 3.0 mmol) and DIEA (516 mg, 4.0 mmol) in CH_2Cl_2 (8 mL) was stirred at room temperature for 4 h. The mixture was diluted with water (100 mL) and extracted with dichloromethane 100 mL×3). The combined organic layer was dried over Na_2SO_4, filtrated and concentrated to give the residue, which was purified by flash column chromatography on silica gel (eluent: DCM/MeOH = 95/5) to give tert-butyl 4-(4-cyclohexylbenzoyl)piperazine-1-carboxylate (500 mg, 67%) as a white solid. MS (ESI) m/z = 373.2 $[M+H]^+$

<u>Step 2: synthesis of (4-cyclohexylphenyl)(piperazin-1-yl)methanone (B-2)</u>

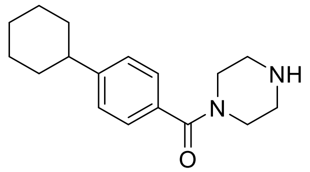

To a solution of tert-butyl 4-(4-cyclohexylbenzoyl)piperazine-1-carboxylate (480.00 mg, 1.29 mmol) in dichloromethane (4 mL) was added 4 N HCl/dioxane (4 mL) and the solution was stirred at room temperature for 2 h. The reaction solution was concentrated under vacuum to give the product (4-cyclohexylphenyl)(piperazin-1-yl)methanone (400.00 mg

HCl salt, crude) as a white solid which was used for next step without further purification.MS (ESI) m/z =273.2 $[M+H]^+$.

<u>Step 3: synthesis of 4-(4-(4-cyclohexylbenzoyl)piperazine-1-carbonyl)benzene sulfonamide (</u>GDI-009108)

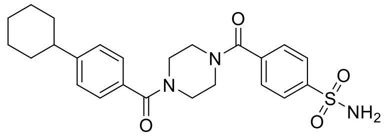

GDI-009108

A solution of 4-sulfamoylbenzoic acid (332.00 mg, 1.65 mmol) in $SOCl_2$ (8 mL) was stirred at 90 degrees for 4 h. After vacuum concentration, the residue was dissolved in CH_2Cl_2 (3 mL). Another bottle in the shape of an eggplant was added (4-cyclohexylphenyl)(piperazin-1-yl)methanone (300.00 mg, 1.10 mmol) and DIEA (569.00 mg, 4.40 mmol) in CH_2Cl_2 (5 mL), then added the above mixture and the reaction mixture was stirred at 25 degrees for 1 h. The mixture was diluted with water (100 mL) and extracted with dichloromethane 100 mL×3). The combined organic layers were dried over Na_2SO_4, filtrated and concentrated to give the residue, which was purified by flash column chromatography on silica gel (eluent: DCM/MeOH = 95/5) to give 4-(4-(4-cyclohexylbenzoyl)piperazine-1-carbonyl)benzenesulfonamide (51 mg, 9.19 %) as a white solid.MS (ESI) m/z = 456.1 $[M+H]^+$.

^{1}H NMR (400 MHz, d6-DMSO) δ 7.88 (d, J = 7.9 Hz, 2H), 7.62 (d, J = 8.0 Hz, 2H), 7.49 (s, 2H), 7.36-7.27 (m, 4H), 3.56-3.35 (m, 8H), 2.54 (s, 1H), 1.81-1.68 (m, 5H), 1.46 – 1.31 (m, 4H), 1.24 (dd, J = 12.7, 8.6 Hz, 1H).

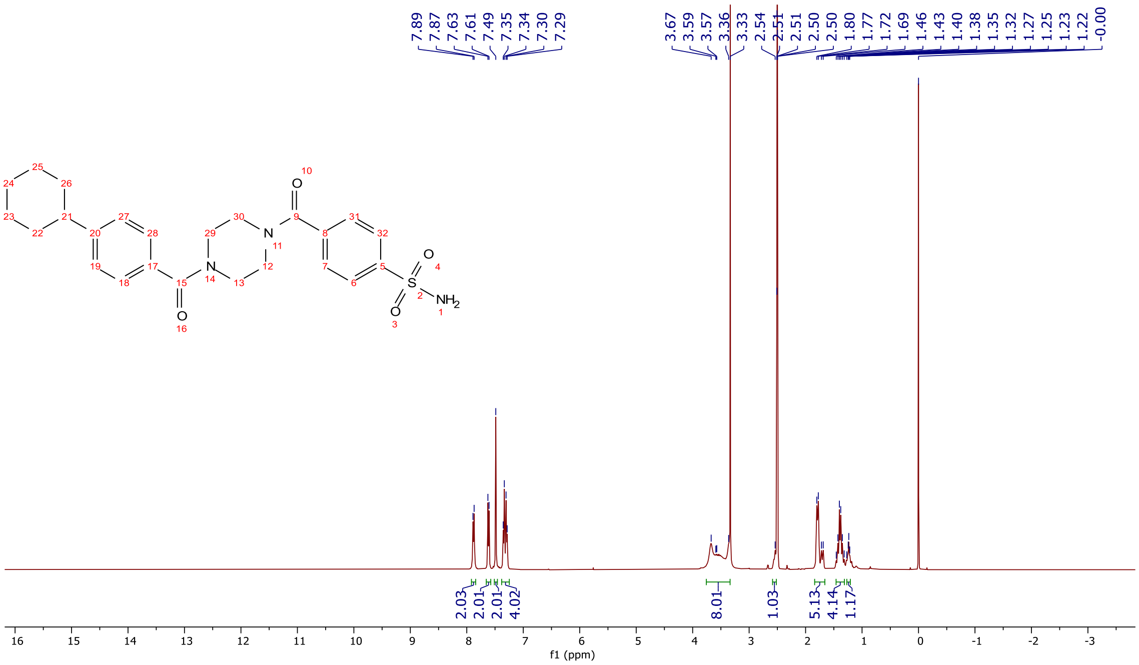

^{1}H NMR of **GDI-009108**

Synthesis of Syn-C4-001 in the SI

The chemists responsible for the synthesis refer to the compound Syn-C4-001 by an internal code GDI-009109.

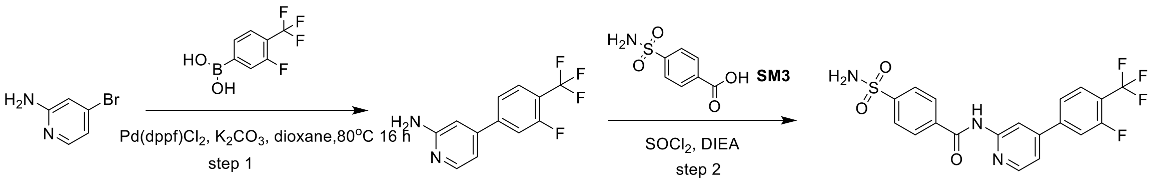

*N-(4-(3-fluoro-4-(trifluoromethyl)phenyl)pyridin-2-yl)-4-sulfamoylbenzamide(**GDI-009109)***

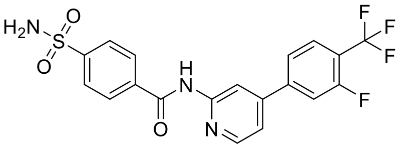

GDI-009109

^{1}H NMR (400 MHz, DMSO) δ 11.27 (s, 1H), 8.56 (dd, *J* = 3.4, 1.1 Hz, 2H), 8.19 (d, *J* = 8.5 Hz, 2H), 8.01 – 7.97 (m, 2H), 7.95 (d, *J* = 8.6 Hz, 2H), 7.83 (d, *J* = 8.4 Hz, 1H), 7.64 – 7.62 (m, 1H), 7.56 (s, 2H).

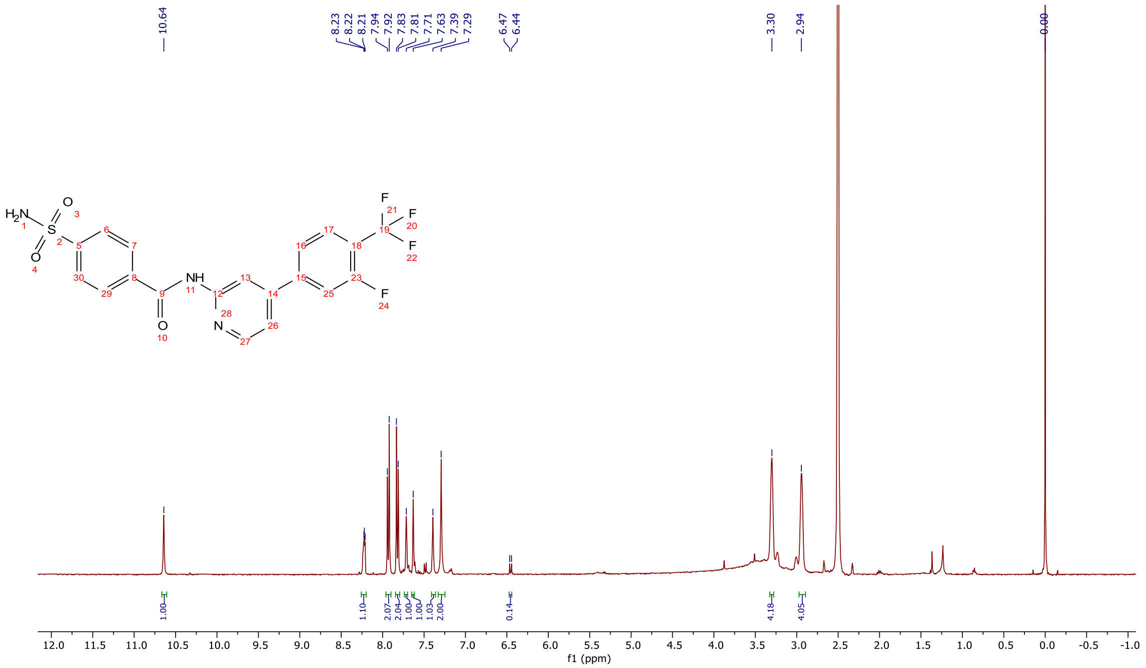

^{1}H NMR of **GDI-009109**

Synthesis of Syn-C5-001 in the SI

The chemists responsible for the synthesis refer to the compound Syn-C5-001 by an internal code GDI-009110.

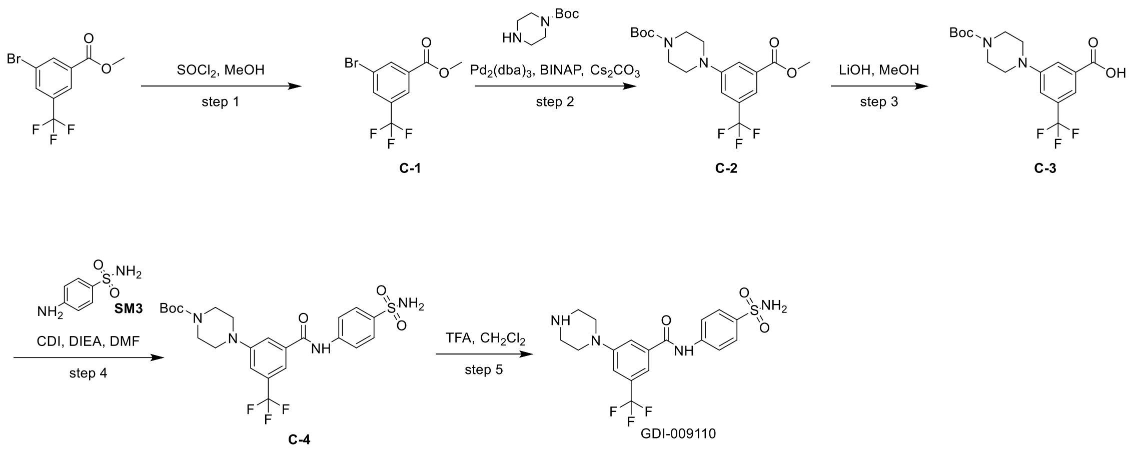

3-(piperazin-1-yl)-N-(4-sulfamoylphenyl)-5-(trifluoromethyl)benzamide(GDI-009110)

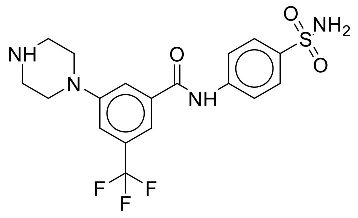

GDI-009110

^{1}H NMR (400 MHz, DMSO) δ 10.64 (s, 1H), 8.26 – 8.20 (m, 1H), 7.93 (d, *J* = 8.8 Hz, 2H), 7.82 (d, *J* = 8.8 Hz, 2H), 7.71 (s, 1H), 7.63 (s, 1H), 7.39 (s, 1H), 7.29 (s, 2H), 3.30 (s, 4H), 2.94 (s, 4H).

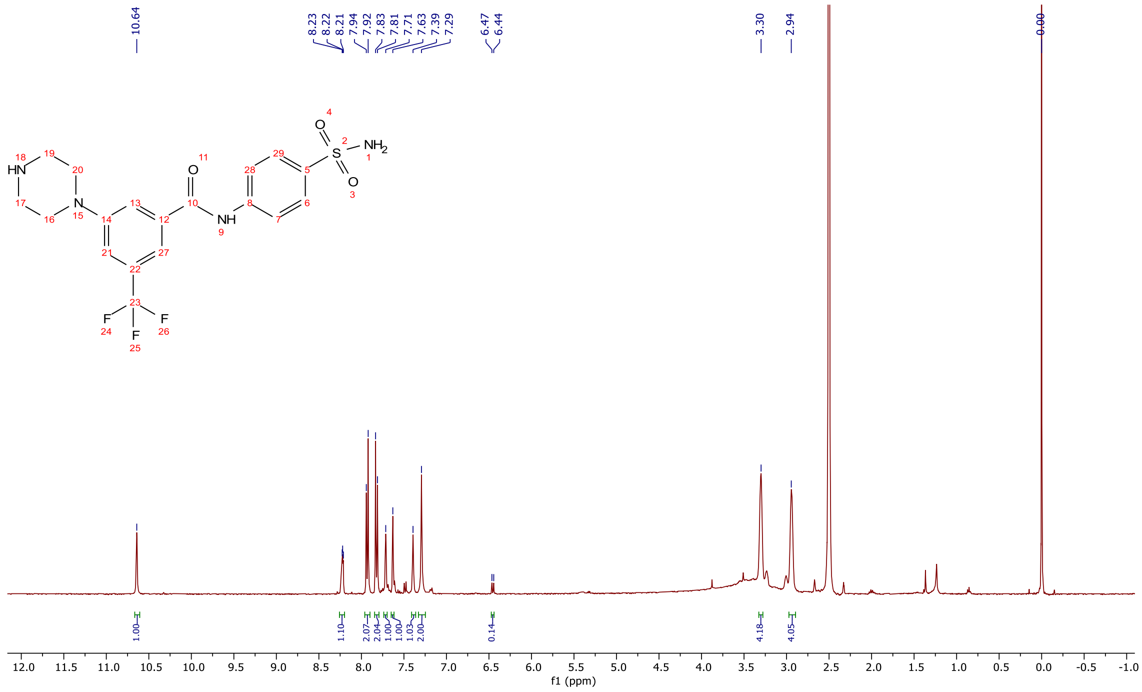

^{1}H NMR of **GDI-009110**

Synthesis of Syn-C6-001 in the SI

The chemists responsible for the synthesis refer to the compound Syn-C6-001 by an internal code GDI-009111

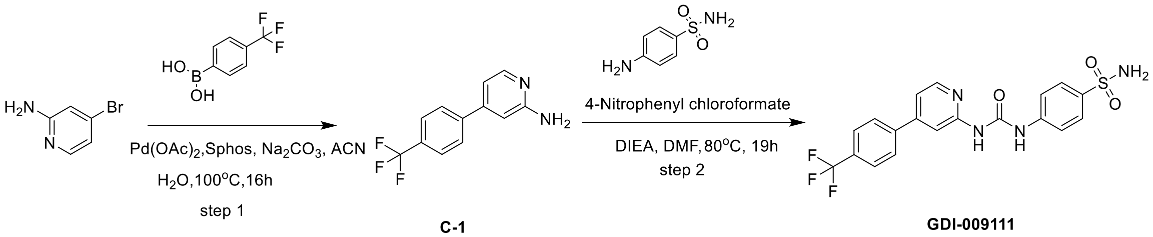

Step 1: synthesis of 4-(4-(trifluoromethyl)phenyl)pyridin-2-amine (C-1)

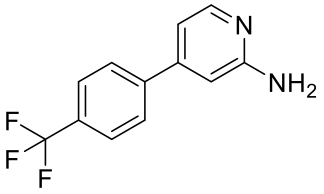

To a stirred mixture of [4-(trifluoromethyl)phenyl]boranediol (2.25 g, 11.8 mmol), 4-bromopyridin-2-amine (2.45 g, 14.2 mmol), Pd(OAc)$_2$ (0.26 g, 1.18 mmol) and S-Phos (0.97 g, 2.36 mmol) in ACN/H_2O (35/35 mL) was added sodium carbonate (2.25 g, 21.2 mmol). The reaction mixture was stirred at 100°C for 16 h under N_2 atmosphere. The reaction mixture was diluted with H_2O (100 mL) and followed by extraction with EtOAc (50 mL × 3). The combined organic layer dried over with anhydrous Na_2SO_4. After filtration, the filtrate was concentrated under vacuum to give the crude product, which was purified by flash column chromatography on silica gel (eluent: DCM/MeOH = 96/4) to give 4-(4-(trifluoromethyl)phenyl)pyridin-2-amine (1.4 g, 45%) as a white solid. MS (ESI) m/z =239.1 $[M+H]^+$.

Step 2: synthesis of 4-(3-(4-(4-(trifluoromethyl)phenyl)pyridin-2-yl)ureido) benzenesulfon amide

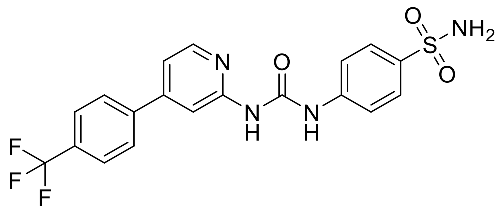

GDI-009111

To a stirred solution of 4-[4-(trifluoromethyl)phenyl]pyridin-2-amine (600 mg, 2.52 mmol) in DMF (10 mL) was added 4-Nitrophenyl chloroformate (507.93 mg, 2.52 mmol). The reaction solution was stirred at 25℃ for 1 h. Then N,N-Diisopropylethylamine (1302.74 mg, 10.08 mmol) and 4-aminobenzenesulfonamide (1093.47 mg, 6.35 mmol) was added to the mixture and the mixture was stirred at 80℃ for 19 h. The reaction mixture was diluted with H_2O (5 mL) and give crude product 150mg. The residue was purified via Genal-Prep-HPLC ((Mobile Phase: ACN-H_20(0.1%TFA) from 55/45 to 30/70 and (Mobile Phase: ACN-H_20(0.1%FA) from 60/40 to 30/70) to give 4-(3-(4-(4-(trifluoromethyl)phenyl)pyridin-2-yl)ureido)benzenesulfon amide (13.7 mg, 1.3%) as a white solid.MS (ESI) m/z =437.0 $[M+H]^+$.

^{1}H NMR (400 MHz, d6-DMSO) δ 10.57 (s, 1H), 9.66 (s, 1H), 8.43 (d, *J* = 5.3 Hz, 1H), 7.99 - 7.86 (m, 5H), 7.78 (d, *J* = 8.9 Hz, 2H), 7.71 (d, *J* = 8.9 Hz, 2H), 7.44 (dd, *J* = 5.3, 1.6 Hz, 1H), 7.25 (s, 2H).

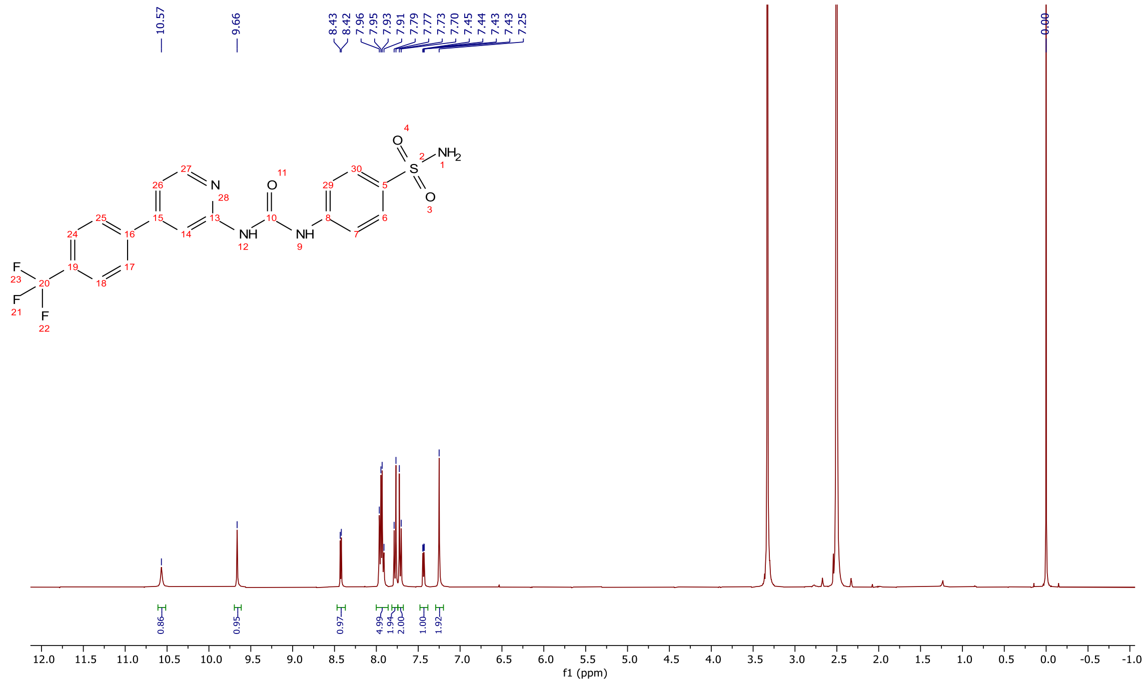

^{1}H NMR of **GDI-009111**

Synthesis of Syn-C7-001 in the SI

The chemists responsible for the synthesis refer to the compound Syn-C7-001 by an internal code GDI-009053

Synthesis of the GDI-009053 (i.e., the Syn-C1-002 in the SI)

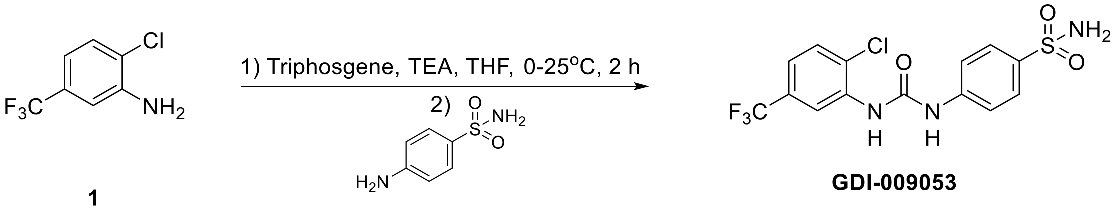

1

GDI-009053

Step 1: Synthesis of 4-(3-(2-chloro-5-(trifluoromethyl)phenyl)ureido) benzene sulfonamide

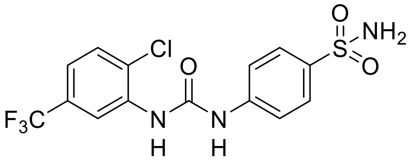

GDI-009053

To a solution of 2 2-chloro-5-(trifluoromethyl)aniline(500.00 mg, 2.54 mmol) and Triphosgene (206.00 mg, 0.76 mmol) in THF (5 mL) at 0℃ was added TEA (772.00 mg, 7.63 mmol) dropwise and stirred at 25℃ for 2 h. Then 4-aminobenzenesulfonamide (481 mg, 2.80 mmol) was added in one charge. The reaction mixture was stirred at 25℃ for 4 h. The resulting mixture was quenching with NH_4Cl and concentrated. The residue was purified by prep-HPLC (Gemini 5 um C18 column, 150*21.2 mm, eluting with 30% to 90% $MeCN/H_2O$ containing 0.1% FA) to afford 4-(3-(2-chloro-5-(trifluoromethyl) phenyl)ureido)benzene sulfonamide (105 mg, 0.26 mmol) as white solid. MS (ESI) m/z = 393.90 $[M+H]^+$

^{1}H NMR (400 MHz, d6-DMSO) δ 9.91 (s, 1H), 8.71 (s, 1H), 8.63 (d, J = 1.5 Hz, 1H), 7.78-7.72 (m, 3H), 7.64 (d, J = 8.8 Hz, 2H), 7.41 (dd, J = 8.4, 1.7 Hz, 1H), 7.24 (s, 2H).

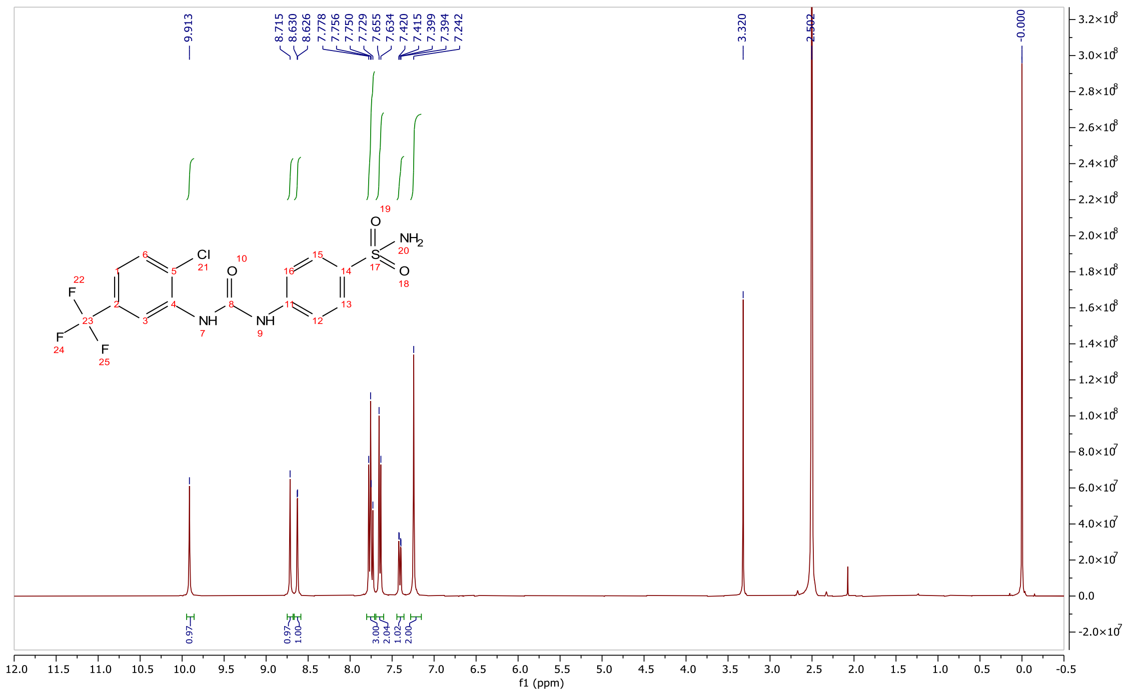

^{1}H NMR of **GDI-009053**

Synthesis of Syn-A003-01

The chemists responsible for the synthesis refer to the compound Syn-A003-01 by an internal code GDI-008111.

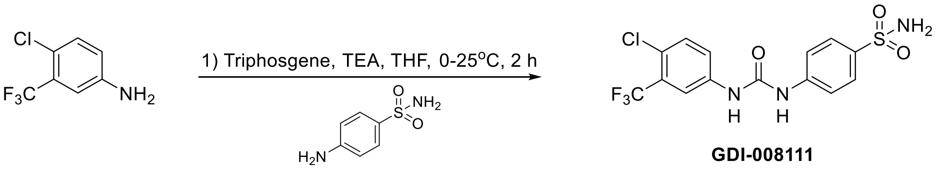

Step 1: Synthesis of 4-(3-(4-chloro-3-(trifluoromethyl)phenyl)ureido)benzenesulfon amide (GDI-008111)

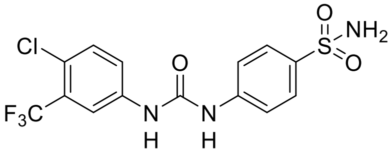

GDI-008111

To a solution of 4-chloro-3-(trifluoromethyl) aniline (500.00 mg, 2.54 mmol) and Triphosgene (206.00 mg, 0.76 mmol) in THF (5 mL) at 0°C was added TEA (772.00 mg, 7.63 mmol) dropwise and stirred at 25°C for 2 h. Then 4-aminobenzenesulfonamide (481.00 mg, 2.80 mmol) was added in one charge. The reaction mixture was stirred at 25°C for 4 h. The resulting mixture was quenching with NH_4Cl and concentrated. The residue was purified by prep-HPLC (Gemini 5 um C18 column, 150*21.2 mm, eluting with 30% to 90% $MeCN/H_2O$ containing 0.1% FA) to afford 4-(3-(4-chloro-3-(trifluoromethyl) phenyl)ureido) benzenesulfonamide (56 mg, 0.14 mmol) as white solid.MS (ESI) m/z = 393.90 $[M+H]^+$

^{1}H NMR (400 MHz, d6-DMSO) δ 9.26 (d, J = 15.1 Hz, 2H), 8.12 (s, 1H), 7.74 (d, J = 8.8 Hz, 2H), 7.63 (d, J = 9.0 Hz, 4H), 7.22 (s, 2H).

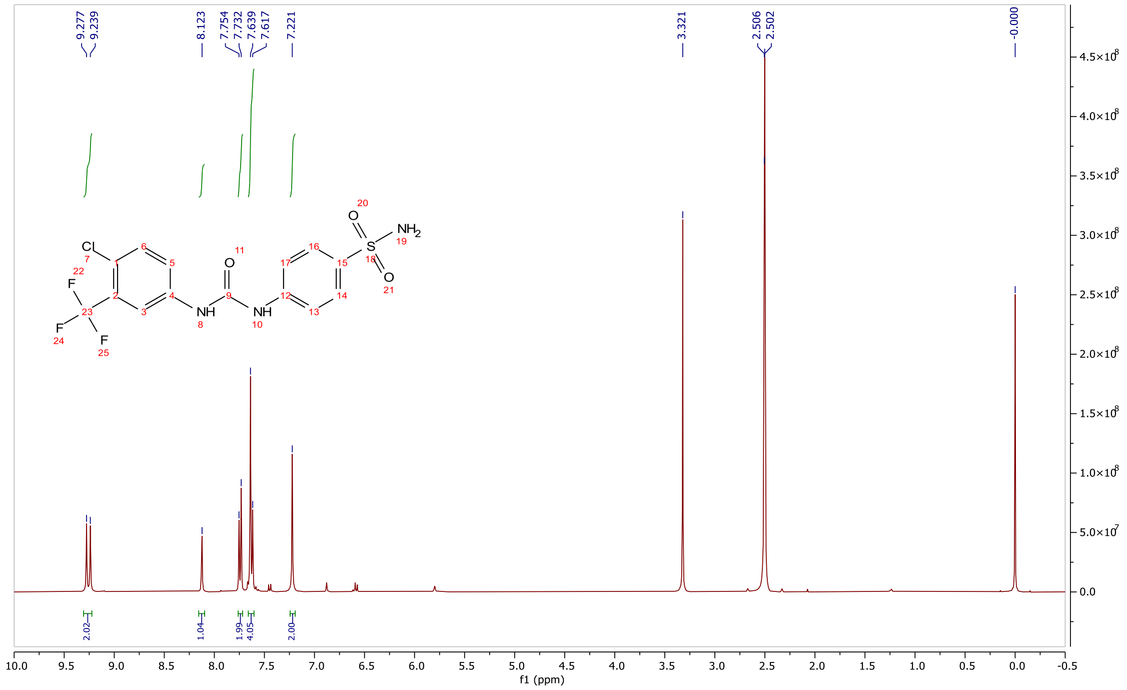

^{1}H NMR of **GDI-008111**
